# Early and Later Perceptions and Reactions to the COVID-19 Pandemic in Germany: On Predictors of Behavioral Responses and Guideline Adherence During the Restrictions

**DOI:** 10.3389/fpsyg.2021.769206

**Published:** 2021-11-26

**Authors:** Eva Lermer, Matthias F. C. Hudecek, Susanne Gaube, Martina Raue, Falk Batz

**Affiliations:** ^1^LMU Center for Leadership and People Management, LMU Munich, Munich, Germany; ^2^Institute of Business Psychology, FOM University of Applied Sciences, Munich, Germany; ^3^Applied Business and Media Psychology program, Ansbach University of Applied Sciences, Ansbach, Germany; ^4^Department of Experimental Psychology, University of Regensburg, Regensburg, Germany; ^5^Department of Infection Prevention and Infectious Diseases, University Hospital of Regensburg, Regensburg, Germany; ^6^MIT AgeLab, Massachusetts Institute of Technology, Cambridge, MA, United States; ^7^Department of Obstetrics and Gynecology and Center for Gynecological Endocrinology and Reproductive Medicine, University Hospital, LMU Munich, Munich, Germany

**Keywords:** COVID-19, trust, behavior change, guideline adherence, social distancing, political orientation, acceptance, health anxiety

## Abstract

In March 2020, the German government enacted measures on movement restrictions and social distancing due to the COVID-19 pandemic. As this situation was previously unknown, it raised numerous questions about people’s perceptions of and behavioral responses to these new policies. In this context, we were specifically interested in people’s trust in official information, predictors for self-prepping behavior and health behavior to protect oneself and others, and determinants for adherence to social distancing guidelines. To explore these questions, we conducted three studies in which a total of 1,368 participants were surveyed (Study 1 *N*=377, March 2020; Study 2 *N*=461, April 2020; Study 3 *N*=530, April 2021) across Germany between March 2020 and April 2021. Results showed striking differences in the level of trust in official statistics (depending on the source). Furthermore, all three studies showed congruent findings regarding the influence of different factors on the respective behavioral responses. Trust in official statistics predicted behavioral responses in all three studies. However, it did not influence adherence to social distancing guidelines in 2020, but in 2021. Furthermore, adherence to social distancing guidelines was associated with higher acceptance rates of the measures and being older. Being female and less right-wing orientated were positively associated with guidelines adherence only in the studies from 2020. This year, political orientation moderated the association between acceptance of the measures and guideline adherence. This investigation is one of the first to examine perceptions and reactions during the COVID-19 pandemic in Germany across 1year and provides insights into important dimensions that need to be considered when communicating with the public.

## Introduction

On Sunday March 22, 2020, Angela Merkel, the German Chancellor, announced that in the fight against the spread of the novel Coronavirus, she and the prime ministers of Germany agreed that public gatherings of more than two people would be prohibited temporarily for 14days (Frankfurter Allgemeine Zeitung, 2020). Movement restrictions and social/physical distancing provisions have never existed before in the Federal Republic of Germany, and so it was unclear how people would react to them. Obviously, the COVID-19 pandemic has raised many questions in many scientific disciplines. Social sciences offered an abundance of theories to predict and explain human behavior in extreme conditions – such as a pandemic. One of the first researchers recommending the application of relevant knowledge from the social and behavioral sciences to the context of the COVID-19 pandemic was Bavel et al. (2020). The extent to which they meet the research zeitgeist is reflected in the number of citations: In October 2021, only about 1.5years after the publication of their article, it has already been cited over 2,400 times. We also wanted to contribute to a better understanding of how people behave in this new situation. Therefore, it was important for us to examine which variables are central for acceptance of the measure and behavioral responses in this context. Based on previous studies in the areas of pandemics (e.g., Ebola: Vinck et al., 2019), prevention measures (e.g., Rykkja et al., 2011), and risk communication (e.g., Baumgartner and Hartmann, 2011), we selected a set of potentially relevant variables. These include trust, political orientation, health anxiety, and uncertainty tolerance.

Like some previous studies (e.g., Longstaff and Yang, 2008; van der Weerd et al., 2011), we consider trust to be an important variable for human behavior in the context of a pandemic. The APA Dictionary of Psychology (2020) defines trust as “reliance on or confidence in the dependability of someone or something.” However, trust is a broad concept and can refer to different aspects, depending on the perspective. The relevant perspective for us at the time of the first study was trust in infection statistics from official authorities, that is, the figures communicated by official institutions and governments. Previous research has shown that trust in political systems may influence people’s reactions to restrictions, that is, trust is positively correlated with acceptance of prevention measures in a society (e.g., anti-terror measures, Rykkja et al., 2011) and linked to law compliance (Marien and Hooghe, 2011). Also, Rowe and Calnan (2006) have shown that trust in public systems and authorities positively influences the way people follow instructions. Greater trust in policy makers is associated with greater compliance in health policies such as testing or quarantining. These relationships could also be demonstrated in past pandemics (e.g., Ebola: Morse et al., 2016; Blair et al., 2017; Asian influenza and H1N1 pandemic: Siegrist and Zingg, 2014). There are some good summaries of the relevance of trust in the context of Coronavirus pandemic (e.g., Balog-Way and McComas, 2020; Devine et al., 2021). Only recently, in the context of the COVID-19 pandemic, it has been shown that trust in institutions is associated with lower mortality rates (Oksanen et al., 2020). Since health authorities used infection and death statistics to justify their strict regulations and encouraged everyone to help “flatten the curve” (of new infections), we expected trust in these official statistics to be an important predictor of compliance with the protective measures. Therefore, we aimed at investigating trust in official information from different sources and formulated the following research question (RQ):

RQ 1: How much do people trust in statistics on COVID-19 from official authorities?

In the course of the COVID-19 pandemic, the media constantly reported about people’s reactions to the new circumstances. This included increased purchasing or even hoarding of products such as disinfectants, face masks, food and toilet paper (Statista, 2020a; Statistisches Bundesamt, 2020), as well as differences in people’s compliance with social distancing measures (Lehrer et al., 2020; Statista, 2020b). Uncertainty about the virus itself, its origin, or the appropriate measures to combat it, coupled with a growing group of people who challenge established facts set the stage for the rise of conspiracy theories. In such an environment, merely trying to convince people of the severity of the disease and the effectiveness of the prevention measures may not be sufficient to encourage protective behavior such as social distancing. Therefore, it is important to not only understand how much people trust in official infection statistics, but also to explore further in the pandemic relevant variables. First, it must be understood which variables are central to behavioral responses to subsequently develop appropriate communication strategies. As behavioral responses, we considered three types of behavior: (A) Self-centered prepping behavior (e.g., stocking up on face masks, food, or other essential goods; the term is also used by Imhoff and Lamberty, 2020, for hoarding everyday goods in the COVID-19 pandemic), and protective behavior to not infect (B) oneself and (C) others. We differentiate here between protective behavior for oneself and for others for different reasons. For example, risk research shows that risk assessments differ depending on who the target person is (i.e., self vs. other, see Lermer et al., 2013, 2019). Furthermore, people differ in prosocial behavior (e.g., Eagly, 2009). While this is more pronounced in some than in others, it need not be related to their self-protective behavior.

Complex and alarming world events are often accompanied by the emergence of conspiracy theories (McCauley and Jacques, 1979; Leman and Cinnirella, 2007; Jolley and Douglas, 2014). These theories assume that the event in question is the result of a secret plot of a powerful group (Imhoff and Bruder, 2014). Previous research suggests that political orientation may be associated with conspiracy beliefs. For instance, van Prooijen et al. (2015) found a positive association between extreme political ideologies (at both sides the right and the left) and the tendency to believe in conspiracy theories. The authors conclude that “political extremism and conspiracy beliefs are strongly associated due to a highly structured thinking style that is aimed at making sense of societal events” (p. 570). A study in Italy has shown that believing in conspiracies is linked to right-wing political orientation (Mancosu et al., 2017). In their recent study in the context of the COVID-19 pandemic, Imhoff and Lamberty (2020) showed that conservative political orientation was positively associated with self-centered prepping behavior (e.g., stocking up on face masks, food, or other essential goods; the term is also used by Imhoff and Lamberty, 2020, for hoarding everyday goods in the COVID-19 pandemic). Due to these findings, we included political orientation in this research. Furthermore, at least two variables seem to be central to behavioral responses during health threatening events: *health anxiety* and *uncertainty tolerance*. Today, numerous studies can be found showing that the COVID-19 pandemic increased levels of anxiety (e.g., Baloran, 2020; Choi et al., 2020; Petzold et al., 2020; Roy et al., 2020; Buspavanich et al., 2021). Fewer studies, however, specifically examine health anxiety and its links to reactions to the COVID-19 pandemic. Research shows that anxiety is linked to safety-seeking behavior (Abramowitz et al., 2007; Tang et al., 2007; Helbig-Lang and Petermann, 2010). For example, health anxiety has been linked to an increase in health information searching (Baumgartner and Hartmann, 2011). Sometimes, however, health anxiety can lead people to avoid relevant information that creates discomfort (Kőszegi, 2003). Avoiding information about a diagnosis, for example, seems to help reduce stress and anxiety, while delaying beneficial action (Golman et al., 2017). In a recent article, Asmundson and Taylor (2020) report that people with high health anxiety also tend to engage in maladaptive behaviors such as panic purchasing. Thus, we were interested in the impact of health anxiety on people’s behavioral responses in the COVID-19 pandemic.

Anxiety is associated with high uncertainty and often motivates people to take action which should reduce uncertainty (Raghunathan and Pham, 1999), such as increased information seeking (Valentino et al., 2009). The COVID-19 pandemic is a threat that is both dreadful and highly uncertain. Research has shown that these affective states strongly influence people’s perceptions of risk (Fischhoff et al., 1978). Perceived risk is influenced by uncertainty (Vives and FeldmanHall, 2018). Uncertainty during the current pandemic is high because SARS-CoV-2 is a novel virus that has until recently not been known to scientists. As a result, it is unclear how the pandemic will develop and difficult to accurately assess one’s personal risk. Uncertainty is a state that is perceived as discomforting and people generally strive to avoid it (Schneider et al., 2017). However, people differ in their tolerance for uncertainty (Grenier et al., 2005). Research on the tolerance of uncertainty goes back to Frenkel-Brunswik (1949) who observed that people systematically differ in dealing with ambiguous situations (Dalbert, 1999). People with a low level of uncertainty tolerance employ vigilant coping strategies such as intensified information seeking about the threatening event. In the context of the COVID-19 pandemic, this could result in reading the news more often than usual. At the same time, people with a low level of uncertainty tolerance tend to show avoidance strategies such as turning away from dreadful information about the threat (Grenier et al., 2005). Thus, we were interested in the impact of uncertainty tolerance on people’s behavioral responses in the COVID-19 pandemic. Furthermore, the variables gender and age seemed important to us to be considered as well. Especially because results of recent studies in the COVID-19 context suggest that these are relevant characteristics regarding behavioral responses. For example, it was shown that women and older participants tended to be more willing to wear face masks (e.g., Capraro and Barcelo, 2020). Also, the results of a study conducted by Li and Liu (2020) suggest that women tend to be engaged in more protective behaviors during the COVID-19 pandemic than men. Furthermore, this also seems to be true for being of older age (Li and Liu, 2020). In sum, we aimed at understanding how trust, political orientation, health anxiety, and uncertainty tolerance, in addition to gender and age, influence people’s self-centered prepping behavior and protective behavior to avoid infection of oneself or others.

RQ 2: How does trust in official statistics, political orientation, health anxiety, uncertainty tolerance, age and gender influence people’s prepping, and health behaviors during the COVID-19 pandemic?

## Study 1 Method

### Participants and Procedure

To explore our RQs, we conducted an online survey end of March, 2020, when the restrictions in Germany started. At that time (March 23), 24,774 people had been infected and 94 had died from or with COVID-19 in Germany (WHO, 2020b). A total of 571 people clicked on the questionnaire. Data from participants who failed to answer the attention check item (i.e., *If you add two and three, you get four*) correctly or did not finish the questionnaire were not included. The final sample encompassed data from 377 participants (78% female) who worked and studied part-time at a university with locations in 32 cities across Germany (*M_age_*=26.54, *SD_age_*=4.97, and *Range_age_*=19–53). There is no information about the individual characteristics of those participants who were excluded. However, all participants originate from the same pool of people (employees or self-employed persons who were enrolled at the FOM University of Applied Sciences as part-time students). One of the participants reported being infected with COVID-19. Forty participants reported that they felt sick, and 10 of those assumed they were infected. All other participants reported that they felt healthy. None of the (possibly) infected participants were excluded. Participants were rewarded with course credits for their participation.

### Measures

#### Trust in Official Infection Statistics

At the time of the survey, media and reporting in Germany mainly covered figures on the incidence of infection from official sources of the governments of China, the EU, Germany, and the Robert Koch Institute (RKI; the RKI is the government’s central scientific institution in the field of biomedicine. It is one of the most important bodies for the safeguarding of public health in Germany.). Trust in statistics from governments and the RKI was measured with four items on a seven-point Likert scale (1=*strongly disagree* to 7=*strongly agree*). The items were all designed in the same way: “*I trust the statistics from … (1) China, (2) European Countries, (3) German government, (4) RKI on the Corona virus*.” These items were averaged to an index of *trust* (Cronbach’s *α*=0.86), which was used as an independent variable to investigate RQ 2.

#### Political Orientation

Participants’ political orientation was measured using the Left–Right Self-Placement scale developed by Breyer (2015). This scale measures political attitudes on a left-right dimension with a single item asking participants to locate themselves on a 10-point Likert scale with the poles left and right.

#### Health Anxiety

Health anxiety was measured using the German version of the health anxiety inventory (MK-HAI) developed by Bailer and Witthöft (2014). This scale assesses the trend toward health-related concerns with 14 items on a five-point Likert scale (1=*strongly disagree* to 5=*strongly agree*); sample item: “I spend a lot of time worrying about my health.” These items were averaged to an index of *health anxiety* (Cronbach’s *α*=0.93).

#### Uncertainty Tolerance

We measured uncertainty tolerance with the Uncertainty Tolerance (UT) Scale developed by Dalbert (1999). This questionnaire captures the tendency to assess uncertain situations as threats or challenges with eight items on a six-point Likert scale (1=*strongly disagree* to 6=*strongly agree*); sample item: “I like to know what to expect.” These items were averaged to an index of *uncertainty tolerance* (Cronbach’s *α*=0.70).

#### Self-Centered Prepping Behavior

Self-centered prepping behavior in the context of COVID-19 was measured using three items: “I bought face masks;” “I stocked up on food;” and “I stocked up on disinfectant.” The answer format was yes or no. Yes answers were summed up to a self-centered prepping behavior sum value. At the time of the study, it was not yet clear (at least to the public) that wearing a mask was more protective for others than for oneself. In addition, masks were a scarce commodity at the time. At the beginning of the Corona pandemic, not even system-relevant institutions (e.g., hospitals) were supplied with sufficient amounts of masks (Biermann et al., 2020; WHO, 2020a). Thus, masks were difficult to obtain at that time. Also, an official requirement to wear masks in public (e.g., while shopping and public transportation) was not introduced throughout Germany until April 29, 2020 (Mitze et al., 2020; The Federal Government Germany, 2020). We therefore understand buying face masks as a behavior to hoard a certain good to build up a stock on them for a certain period of time. With this understanding, we follow the conceptualization of self-prepping behavior described by Imhoff and Lamberty (2020).

#### Protective Behavior for Self-Protection

Protective behavior to avoid infection was measured using four items. Individuals were asked to indicate change in behavior or new behavior on a five-point Likert scale (*1*=*strongly disagree* to *5*=*strongly agree*). Items were: “I avoid public transport (contact with other people/visiting cafés and restaurants/meetings with friends) in order not to get infected.” These items were averaged to an index of *behavior change for self-protection* (Cronbach’s *α*=0.81).

#### Protective Behavior for Others

The protective behavior to not infect others was measured using the same four items as to measure behavior change for self-protection. However, these items were related to other people; a sample item reads, “I avoid public transport to protect others.” These items were averaged to an index of *behavior change for others* (Cronbach’s *α*=0.88).

## Study 1 Results

To answer RQ 1, participants’ trust in statistics on COVID-19 infections from different official authorities was analyzed, and the results are shown in Table 1. Trust in statistics from China was by far the lowest, whereas trust in statistics from the RKI was highest.

**Table 1:** Participants’ trust in official information in Study 1, Study 2, and Study 3.

*I trust the statistics from*	Study 1 (*N*=377) March 2020 *M* (*SD*)	Study 2 (*N*=461) April 2020 *M* (*SD*)	Study 3 (*N*=530) April 2021 *M* (*SD*)
China	2.89 (1.58)	2.23 (1.36)	2.28 (1.38)
European Countries	4.28 (1.67)	3.83 (1.66)	3.95 (1.73)
German Government	4.85 (1.72)	4.63 (1.76)	4.53 (1.88)
Robert Koch Institute	5.22 (1.64)	4.94 (1.76)	4.89 (1.90)

The scale ranged from 1=strongly disagree to 7=strongly agree.

To answer RQ 2, we analyzed associations with behavioral responses by using correlation analyses. Results can be found in Table 2.

**Table 2:** Means, SDs, and correlations for gender, age, trust in official information, political orientation, health anxiety and uncertainty tolerance with self-centered prepping behavior, behavior change to avoid infection, and behavior change in order to not infect others in Study 1.

	Prepp. beh.	BC for self	BC for others
1. Gender	0.008	**0.149** ^**^	**0.197** ^***^
2. Age	0.101	**0.112** ^*^	−0.031
3. Trust Info.	**−0.114** ^*^	**0.146** ^**^	**0.100** ^**#**^
4. Left–Right	0.027	**−0.116** ^*^	**−0.120** ^*^
5. MK-HAI	**0.127** ^*^	**0.092** ^**#**^	0.059
6. UT	0.001	−0.048	−0.091
Total: *M* (*SD*)	0.58 (0.74)	4.38 (0.75)	4.35 (0.88)
Men: *M* (*SD*)	0.55 (0.82)	4.13 (0.93)	4.05 (1.01)
Women: *M* (*SD*)	0.58 (0.82)	4.45 (0.69)	4.43 (0.81)
*t*-Test	0.42	**2.76** ^**^	**3.10** ^**^
Cohen’s *d*	0.03	0.39	0.41

Gender: 1=male, 2=female; Trust Info. = Trust in official information (Range=1–7), higher values indicate more trust; Left–Right = political orientation (Range=1–10), higher values indicate more right orientation; MK-HAI = Health anxiety (Range=1–5), higher values indicate more anxiety; UT = Uncertainty tolerance (Range=1–6), higher values indicate more uncertainty tolerance; Prepp. beh. = Self-centered prepping behavior (Study 1: Range=0–3), higher values indicate more self-centered prepping behavior; BC for self = behavior change for self-protection/to avoid infection (Range=1–5), higher values indicate more behavior change; and BC for others = Behavior change in order to not infect others (Range=1–5), higher values indicate more behavior change.

# indicates *p*<0.10.

* indicates *p*<0.05;

** indicates *p*<0.01;

*** indicates *p*<0.001. Bold highlighted values are at least *p*<0.10.

All variables, except uncertainty tolerance, showed significant associations with one of the three behavioral responses. To investigate the role of these variables to predict behavior change, we conducted linear multiple regression analyses. Findings are shown in Table 3.

**Table 3:** Multiple linear regressions on self-centered prepping behavior, behavior change to avoid infection, and behavior change in order to not infect others in Study 1.

	Study 1				
	Unstandardized coefficients		Standardized coefficients		
	*B*	*SE*(*B*)	*β*	*t*	*p*
**Prepp. beh.**
(Constant)	0.09	0.34		0.28	0.778
Gender	0.04	0.09	0.02	0.44	0.657
Age	0.01	0.00	0.07	1.48	0.137
Trust Info.	−0.05	0.02	**−0.10** ^*^	2.02	0.044
Left–Right	0.00	0.02	0.02	0.40	0.688
MK-HAI	0.12	0.04	**0.13** ^*^	2.52	0.012
*adj. R^2^*	0.024				
*F*	**2.80** ^*^				0.017
**BC for self**					
(Constant)	2.97	0.35		8.51	0.000
Gender	0.29	0.09	**0.15** ^**^	3.10	0.002
Age	0.01	0.00	**−0.11** ^*^	2.32	0.021
Trust Info.	0.08	0.02	**0.14** ^**^	2.91	0.004
Left–Right	−0.03	0.02	−0.07	1.45	0.136
MK-HAI	0.08	0.05	**0.08** ^**#**^	1.70	0.089
*adj. R^2^*	0.064				
*F*	6.04^***^				0.000
**BC for others**					
(Constant)	3.55	0.40		8.72	0.000
Gender	0.35	0.11	**0.16** ^**^	3.23	0.001
Age	0.00	0.00	0.00	0.12	0.904
Trust Info.	0.05	0.32	**0.09** ^**#**^	1.77	0.077
Left–Right	−0.05	0.02	**−0.09** ^**#**^	1.80	0.073
MK-HAI	0.43	0.05	0.03	0.73	0.463
*adj. R^2^*	0.039				
*F*	3.94^**^				0.002

Gender: 1=male, 2=female; Trust Info.=trust in official information (Range=1–7), higher values indicate more trust; Left–Right=political orientation (Range=1–10), higher values indicate more right orientation; MK-HAI=health anxiety (Range=1–5), higher values indicate more anxiety; Prepp. Beh.=self-centered prepping behavior (Study 1: Range=0–3; Study 2: Range=1–7), higher values indicate more self-centered prepping behavior; BC for self=behavior change for self-protection/to avoid infection (Range=1–5), higher values indicate more behavior change; and BC for others=behavior change in order to not infect others (Range=1–5), higher values indicate more behavior change.

# indicates *p*<0.10.

* indicates *p*<0.05;

** indicates *p*<0.01;

*** indicates *p*<0.001. Bold highlighted values are at least *p*<0.10.

Results show that lower levels of trust and higher levels of health anxiety are associated with more prepping behavior. Higher levels of trust in official statistics, being female and being of younger age within our sample were shown to be significant predictors for self-protecting behavior. There was also a tendency of higher levels of health anxiety to predict behavior change to avoid infections, which did not reach the significance threshold of *p*<0.05 (*p*=0.09). In the third model, being female was significantly associated with behavior change to not infect others. Furthermore, this latter model also indicated a tendency of higher levels of trust in official statistics and more right-oriented participants to be less likely to change their behavior to protect others. However, these results did not reach the significance threshold of 0.05 (trust: *p*=0.08; political orientation: *p*=0.07).

## Study 1 Discussion

Six major findings arise from Study 1: (a) Trust in official statistics from different authorities depended on the source of the statistics: Data from China were believed much less than data from Europe or Germany. Data from the RKI were most trusted. (b) Trust in official statistics was negatively correlated with self-centered prepping behavior, but positively correlated with behavior to protect oneself and others. This is also in line with other studies showing that trust in institutions of the political system is positively linked to law compliance (e.g., Marien and Hooghe, 2011). Moreover, the public health recommendations mostly focused on hygiene behavior to avoid infections rather than self-centered prepping behavior. In other words, by showing less self-prepping behavior and more of the recommended protective behavior, participants complied with the official recommendations, which may explain why trust decreased self-prepping behavior. Furthermore, these results are in line with a recently conducted study in which social trust (trust in others) was negatively linked to self-prepping behavior during the COVID-19 pandemic (Oosterhoff and Palmer, 2020). (c) Health anxiety predicted both self-centered prepping behavior and behavior change to protect oneself. Research has shown that anxiety leads to actions to reduce uncertainty (Raghunathan and Pham, 1999), and both self-centered prepping behavior and recommended behavior changes (e.g., hygiene behavior) may serve this purpose among individuals with high health anxiety. Furthermore, anxiety has been repeatedly linked to general hoarding behavior (Coles et al., 2003; Timpano et al., 2009), and trait anxiety has also been positively linked to preventative behavior during the COVID-19 pandemic (e.g., avoiding going out and avoiding physical contact; Erceg et al., 2020). (d) Women were more likely to change their behavior to protect both themselves and others. Women not only tend to judge risks as higher than men (e.g., Slovic, 1999) but also engage more in caring behavior (e.g., Archer, 1996) and show more safety-seeking than men (Byrnes et al., 1999; Lermer et al., 2016a; Raue et al., 2018). However, it is important to note that safety behavior may also increase health anxiety (Olatunji et al., 2011), which suggest a potential bidirectional effect. (e) Participants with right-wing political orientations were less likely to change their behavior to protect others.

In sum, these findings not only show differences in people’s trust in official statistics depending on their source but also that trust influences their behavior. These study results demonstrate that trust gained through clear and transparent information and communication of public authorities is a key to decrease uncertainty, limit the spread of false beliefs, and encourage behavior change to protect everyone’s health. A limitation of Study 1 is that we used a dichotomous answer format to assess participants prepping behavior. Furthermore, we did not measure explicitly the trust in government, acceptance of social distancing measures, and guideline adherence. Therefore, a follow-up study was planned where we would assess self-centered prepping behavior in a more detailed way. The aim was to reinvestigate the found correlations and to additionally include the variables trust in government, acceptance of social distancing measures, and guideline adherence and by this expanding the insights gained from Study 1. Herewith, we wanted to follow the call for replication-extension studies (Bonett, 2012; Wingen et al., 2020).

## Study 2 Method

As the COVID-19 pandemic progressed, the duration of the government’s restrictions was extended. To underpin our findings from Study 1, and to further explore the development of perceptions and reactions to the pandemic related restrictions, we replicated and extended Study 1. In addition to reinvestigating our three research questions, we addressed *trust in the government* as well as *acceptance of* and *adherence to social distancing guidelines*.

Trust in authorities is an important factor for the acceptance of many measures and is therefore particularly worth protecting and enhancing (Betsch et al., 2020d). As mentioned above, Rykkja et al. (2011) found that trust in political systems influences citizens’ attitude toward prevention measures. Research from previous epidemics showed that people who had less trust in the government took fewer precautions against the Ebola virus disease during the 2014–2016 outbreak in Liberia and Congo (Vinck et al., 2019; Oksanen et al., 2020). Furthermore, the social development at that time showed that acceptance of government measures *per se* is a particularly relevant variable.

During the pandemic, the media increasingly reported violations of the health protective measures, and the closure of businesses, which led to high rates of unemployment. Around mid-April, people started demonstrating against the measures (Kölner Stadt-Anzeiger, 2020). The behavior of participants in demonstrations against the current measures showed that acceptance of the measures has a strong influence on adherence to social distancing guidelines. Thus, we assessed participants’ trust in the government and acceptance of measures and raised the following research question:

RQ 3: Which factors influence adherence to social distancing guidelines?

To explore this RQ, we analyzed the impact of the variables relevant for behavior change from Study 1, as well as trust in government and acceptance of measures on adherence to social distancing guidelines.

### Participants and Procedure

Our second online survey was conducted between April 8 and April 23, 2020. For the recruitment of participants, we used the same sampling strategy as in Study 1 – only the attention check item was changed. Again, data from participants who failed to answer the attention check item (i.e., *If you would like to continue with this study then select “agree”*; which was the fourth of five response options) correctly or did not finish the questionnaire were not included. We changed the attention check item in comparison to Study 1 because it seemed more valid. In the first study, the attention check was passed by clicking on the rightmost answer option. Here, however, participants could also have passed the check by showing a pattern when answering, as always by clicking on the rightmost answer option. A total of 495 people clicked on the questionnaire. As in the previous study, we only included data from participants who did complete the questionnaire and who passed the attention check. Again, no information about the individual characteristics of the excluded persons is available, but all participants originate from the same pool of people. The remaining sample consisted of 461 (75% female) employed part-time students from the same university with locations in 32 cities across Germany as in Study 1 (*M_age_*=26.45, *SD_age_*=4.05, *Range_age_*=20–49). In Study 2, participants also entered the first digit of their postal code. Data showed that the participants came from all nine postal code areas in Germany. However, the distribution was not equally distributed with most participants living in postal code area four and five where most of the locations (17 out of 32) of the university are (number of participants from area 0=6; 1=21; 2=57; 3=17; 4=177; 5=83; 6=46; 7=9; 8=79; 9=26). Three of the participants reported being infected with COVID-19. Fifteen participants reported that they were sick, and four of those assumed that they were infected. All other participants reported that they were healthy. As in Study 1, all participants remained in the data set independent of their COVID-19 status. Participants were rewarded with course credits for their participation.

### Measures

We applied the same measures for *trust in official statistics* (Cronbach’s *α*=0.85), *political orientation*, *health anxiety* (Cronbach’s *α*=0.93), and *uncertainty tolerance* (Cronbach’s *α*=0.70) as in Study 1. This also applies to the indices *behavior change to avoid infection* (Cronbach’s *α*=0.79) and *behavior change to not infect others* (Cronbach’s *α*=0.80). However, the item “I avoid visiting cafés and restaurants in order not to get infected [/not infect others]” was changed to “I pay more attention to the recommended hygiene rules than before the Coronavirus became known, in order not to get infected [/not infect others]” due to the lockdown.

#### Self-Centered Prepping Behavior

In Study 2, we assessed self-centered prepping behavior in a more detailed way. In order address some limitations of the first study, a Likert scale was used instead of a dichotomous response format and a symmetrical formulation of the items (“purchased” instead of “stocked up” and “bought”). In addition, three more items were developed to examine a wider range of behaviors (e.g., buying hygiene products or disposable gloves). In total, we used six items where participants were asked to indicate on a seven-point Likert scale (1=*strongly disagree* to 7=*strongly agree*), how much each statement applied to them: “Purchased face masks” and “Purchased larger quantities of food [disinfectants/toilet paper/hygiene products/disposable gloves] than usual.” These items were averaged to an index of *self-centered prepping behavior* (Cronbach’s *α*=0.79).

#### Trust in the Government

Participants’ trust in the government was assessed using two items “I have great trust in the federal government” and “I have great trust in the state government” with a seven-point Likert scale (*1*=*strongly disagree* to 7=*strongly agree*) answer format. These items were averaged to an index of *trust in government* (Cronbach’s *α*=0.91). Due to Germany’s federal structure, we surveyed trust in the federal government and state government separately – as did the COVID-19 Snapshot Monitoring (COSMO) project, which is a well-known repeated cross-sectional monitoring project during the COVID-19 outbreak in Germany (see for instance COSMO COVID-19 Snapshot Monitoring, 2020).

#### Acceptance of the Measures

To assess participants’ acceptance of safety measures, four items with a seven-point Likert scale (*1*=*strongly disagree* to *7*=*strongly agree*) answer format were developed: “I think the current measures taken by the German government to combat the COVID-19 pandemic are good,” “I think the German government’s communication of current measures to combat the COVID-19 pandemic is good,” “I think the current measures taken by the federal government to combat the COVID-19 pandemic are appropriate,” and “I think that the people responsible for planning and implementing the current measures have the necessary competence.” These items were averaged to an index of *acceptance* (Cronbach’s *α*=0.89).

#### Adherence to the Social Distancing Guidelines

To assess participants’ adherence to the social distancing guidelines, five items were adapted from a measure of behavior during the COVID-19 pandemic by Rossmann et al. (2020): Participants were asked to indicate on a five-point Likert scale (*1*=*never* to *5*=*very often*) how often in the last 10days the following applied to them: “I met with friends who live outside my household;” “I met with family members who live outside my household;” “I met with older people;” “I violated the 1.5 meters distance rule;” and “I disregarded regulations on social distancing or movement restrictions.” These items were averaged to an index of *guideline adherence* (Cronbach’s *α*=0.64) and recoded so that higher values indicate more adherence.

## Study 2 Results

As in Study 1, to answer RQ 1, people’s level of trust in statistics on COVID-19 from official authorities was compared and displayed in Table 1. Again, findings show that trust in statistics from China was by far lowest, whereas trust in statistics from the RKI was highest.

To reinvestigate RQ 2, correlation analyses from Study 1 were replicated and presented in Table 4. Whereas in Study 1, all variables except uncertainty tolerance showed significant correlations with behavior change, and in Study 2, all variables showed significant links with at least one behavior variable.

**Table 4:** Means, SDs, and correlations for gender, age, trust in official information, political orientation, health anxiety and uncertainty tolerance with self-centered prepping behavior, behavior change to avoid infection and behavior change in order to not infect others in Study 2.

	Prepp. beh.	BC for self	BC for others
1. Gender	0.029	0.064	**0.180** ^***^
2. Age	**−0.080** ^**#**^	−0.018	0.016
3. Trust Info.	−0.055	**0.136** ^**^	**0.148** ^**^
4. Left–Right	0.024	−0.003	**−0.130** ^**^
5. MK-HAI	**0.168** ^***^	**0.133** ^**^	0.031
6. UT	0.000	**−0.105** ^*^	−0.046
Total: *M* (*SD*)	2.05 (1.11)	4.05 (0.85)	4.13 (0.88)
Men: *M* (*SD*)	1.99 (1.23)	3.97 (0.85)	3.86 (0.95)
Women: *M* (*SD*)	2.07 (1.07)	4.08 (0.83)	4.22 (0.83)
*t*-Test	0.61	1.10	3.84^***^
Cohen’s *d*	0.06	0.13	0.40

Gender: 1=male, 2=female; Trust Info.=trust in official information (Range=1–7), higher values indicate more trust; Left–Right=political orientation (Range=1–10), higher values indicate more right orientation; MK-HAI=health anxiety (Range=1–5), higher values indicate more anxiety; UT=uncertainty tolerance (Range=1–6), higher values indicate more uncertainty tolerance; Prepp. beh.=self-centered prepping behavior (Study 1: Range=0–3; Study 2: Range=1–7), higher values indicate more self-centered prepping behavior; BC for self=behavior change for self-protection/to avoid infection (Range=1–5), higher values indicate more behavior change; BC for others=behavior change in order to not infect others (Range=1–5), higher values indicate more behavior change.

# indicates *p*<0.10.

* indicates *p*<0.05;

** indicates *p*<0.01;

*** indicates *p*<0.001. Bold highlighted values are at least *p*<0.10.

For comparison reasons, the same variables as in Study 1 were included in multiple regressions on the dependent variables of self-centered prepping behavior, behavior change to avoid infection, and behavior change in order to not infect others (see Table 5). Again, results showed that health anxiety was positively associated with self-centered prepping behavior and behavior change to avoid infection of one self. Further, the results again showed that trust in official statistics was positively associated with behavior change to avoid infections of oneself and others. Additionally, behavior change to not infect others was further predicted by being female and less right-oriented, which mirrors the pattern of Study 1.

**Table 5:** Multiple linear regressions on self-centered prepping behavior, behavior change to avoid infection, and behavior change in order to not infect others in Study 2.

	Study 2				
	Unstandardized coefficients		Standardized coefficients		
	*B*	*SE*(*B*)	*β*	*t*	*p*
**Prepp. beh.**
(Constant)	0.97	0.50		1.93	0.054
Gender	0.04	0.12	0.01	0.34	0.730
Age	0.02	0.01	**0.08** ^**#**^	1.85	0.064
Trust Info.	−0.05	0.06	−0.06	1.42	0.156
Left–Right	0.00	0.03	0.00	0.14	0.887
MK-HAI	0.25	0.06	**0.17** ^***^	3.77	0.000
*adj. R^2^*	0.030				
*F*	3.76^**^				0.002
**BC for self**					
(Constant)	3.34	0.38		8.78	0.000
Gender	0.09	0.09	0.05	1.05	0.291
Age	−0.01	0.01	−0.00	−0.08	0.929
Trust Info.	0.06	0.02	0.10^*^	2.29	0.022
Left–Right	0.00	0.02	0.00	1.59	0.874
MK-HAI	0.12	0.05	0.11^*^	2.46	0.014
*adj. R^2^*	0.020				
*F*	2.88^*^				0.014
**BC for others**					
(Constant)	3.19	0.39		8.17	0.000
Gender	0.35	0.09	**0.17** ^***^	3.67	0.000
Age	0.07	0.01	0.03	0.72	0.472
Trust Info.	0.08	0.03	**0.13** ^**^	2.93	0.004
Left–Right	−0.05	0.02	**−0.09** ^*^	2.01	0.044
MK-HAI	0.01	0.05	0.00	0.19	0.845
*adj. R^2^*	0.050				
*F*	5.82^***^				0.000

Gender: 1=male, 2=female; Trust Info.=trust in official information (Range=1–7), higher values indicate more trust; Left-Right=political orientation (Range=1–10), higher values indicate more right orientation; MK-HAI=health anxiety (Range=1–5), higher values indicate more anxiety; Prepp. Beh.=self-centered prepping behavior (Study 1: Range=0–3; Study 2: Range=1–7), higher values indicate more self-centered prepping behavior; BC for self=behavior change for self-protection/to avoid infection (Range=1–5), higher values indicate more behavior change; and BC for others=behavior change in order to not infect others (Range=1–5), higher values indicate more behavior change.

# indicates *p*<0.10.

* indicates *p*<0.05;

** indicates *p*<0.01;

*** indicates *p*<0.001. Bold highlighted values are at least *p*<0.10.

To investigate who shows more adherence to social distancing guidelines and answer RQ 3, correlations of guideline adherence with relevant variables from Study 1 (gender, age, trust in official statistics, political orientation, and health anxiety) as well as acceptance of the measures and trust in government were analyzed in a first step. The correlation matrix can be found in Table 6.

**Table 6:** Means, SDs, and correlations for gender, age, trust in official information, political orientation, health anxiety, acceptance of the measures, and trust in the government with adherence to the social distancing guidelines in Study 2.

	Guideline adherence
1. Gender	**0.134** ^**^
2. Age	**0.120** ^**^
3. Trust Info.	**0.092** ^*^
4. Left–Right	**−0.110** ^*^
5. MK-HAI	−0.054
6. Acceptance	**0.143** ^**^
7. Trust Gov.	0.120
Total: *M* (*SD*)	4.08 (0.62)
Men: *M* (*SD*)	3.93 (0.61)
Women: *M* (*SD*)	4.41 (0.62)
*t*-Test	**2.93** ^**^
Cohen’s *d*	0.39

Gender: 1=male, 2=female; Trust Info.=trust in official information (Range=1–7), higher values indicate more trust; Left-Right=political orientation (Range=1–10), higher values indicate more right orientation; MK-HAI=health anxiety (Range=1–5), higher values indicate more anxiety; Acceptance (Range=1–7), higher values indicate more acceptance of the measures; Trust Gov.=trust in government (Range=1–7), higher values indicate more trust in the government; and Guideline adherence=adherence to the social distancing guidelines (Range=0–5), higher values indicate more adherence.

* indicates *p*<0.05;

** indicates *p*<0.01. Bold highlighted values are at least p<0.10.

All variables except health anxiety and trust in government showed significant correlations with guideline adherence. Thus, all variables showing significant links were included as predictors in a multiple linear regression with guideline adherence as the dependent variable. Findings are presented in Table 7. Results show that adherence to the social distancing guidelines was positively associated with higher levels of acceptance of the measures, being female and being of older age. There was also a tendency of more right-oriented participants to adhere less to social distancing guidelines (*p*<0.10).

**Table 7:** Multiple linear regressions on adherence to the social distancing guidelines.

	Study 2				
	Unstandardized coefficients		Standardized coefficients		
	*B*	*SE*(*B*)	*β*	*t*	*p*
**Guid. Adh.**
(Constant)	3.04	0.28		10.68	0.000
Gender	0.20	0.06	**0.13** ^**^	2.97	0.003
Age	0.02	0.00	**0.13** ^**^	2.94	0.003
Trust Info.	0.01	0.02	0.02	0.44	0.656
Left–Right	−0.03	0.01	**−0.07** ^**#**^	1.68	0.094
Acceptance	0.06	0.02	**0.12** ^*^	2.31	0.021
*adj. R^2^*	**0.053**				
*F*	**6.07** ^***^				0.000

Guid. Adh.=adherence to the social distancing guidelines (Range=0–5), higher values indicate more adherence, Gender: 1=male, 2=female; Trust Info.=trust in official information (Range=1–7), higher values indicate more trust; Left-Right=political orientation (Range=1–10), higher values indicate more right orientation, Acceptance, acceptance of the measures (Range=1–7), higher values indicate more acceptance.

# indicates *p*<0.10.

* indicates *p*<0.05;

** indicates *p*<0.01;

*** indicates *p*<0.001. Bold highlighted values are at least *p*<0.10.

We also conducted a moderation analysis to test whether political orientation is a moderator of the relationship between acceptance of the measures and guideline adherence. We used Hayes’ PROCESS tool (model 1). Results showed a significant interaction effect of measure acceptance and political orientation (*B*=0.03, *SE*=0.01, *t*=2.46, 95%-CI=[0.01; 05]). Analyses of conditional effects revealed no relationship between measure acceptance and guideline adherence (*B*=0.02, *SE*=0.03, *t*=0.53, 95%-CI=[−0.05; 0.07]) for less right-wing orientated participants (1 *SD* below the mean). For participants with average values (mean centered; *B*=0.08, *SE*=0.02, *t*=3.25, 95%-CI=[0.02; 0.11]) and for those with more right-wing orientation (1 *SD* above the mean; *B*=0.11, *SE*=0.03, *t*=3.77, 95%-CI=[0.05; 0.17]), results showed a significant relationship between acceptance of the measures and guideline adherence. Political orientation was non-normally distributed, with skewness of 0.08 (*SE*=0.11) and kurtosis of 0.09 (*SE*=0.22), indicating a right-skewed left-leaning distribution. The average value was *M*=4.53 (*SD*=0.08) slightly below the mean of the scale, the median=5. Moreover, while analyses for gender showed no moderation effect (*p*=0.869), we found that age was a moderator for the effect of acceptance of measures on guideline adherence (*B*=0.02, *SE*=0.01, *t*=2.35, CI-95%=[0.00; 03]). Analyses of conditional effects revealed no relationship between measure acceptance and guideline adherence (*B*=0.03, *SE*=0.03, *t*=0.83, 95%-CI=[−0.03; 0.09]) for participants aged around 23years. For participants aged around 25years (*B*=0.06, *SE*=0.02, *t*=2.30, 95%-CI=[0.01; 0.10]) and for those aged around 29years (*B* =0.11, *SE*=0.03, *t*=3.95, 95%-CI=[0.06; 0.17]), results showed a significant relationship between acceptance of the measures and guideline adherence.

## Study 2 Discussion

Study 2 successfully replicated the findings from Study 1: (a) In the further course of the pandemic, there were still differences in trust in official statistics from different authorities: Again, data from China were believed much less than data from Europe or Germany, whereas data from the RKI were most trusted. (b) As in Study 1, results showed that health anxiety increases self-centered prepping behavior and behavior change to avoid infections. Also, trust in official statistics increased behavior change to avoid infections. Replicating the findings from Study 1, results from Study 2 indicate that being female, being less politically right-oriented, and having trust in official statistics increases behavior change in order to not infect others.

In addition to replicating findings from Study 1, Study 2 aimed at investigating influences on adherence to social distancing guidelines. Results show that guideline adherence was positively associated with older age, being female, less right-wing political orientation, and higher acceptance of the measures. A recently conducted study on guideline adherence during the pandemic in the United States also reports a small positive relationship with age (Bogg and Milad, 2020). However, the authors did not show a significant association with gender. Findings from previous research do however support the assumption that women tend to show more precautionary behaviors to avoid infections. For instance, studies show women generally practice more frequent hand-washing than men (Liao et al., 2010; Park et al., 2010). Furthermore, findings from a meta-analysis (Moran and Del Valle, 2016) indicate inherent differences in how women and men respond to pandemic diseases: women are more likely to practice preventative behavior (e.g., face mask wearing) and avoidance behavior (e.g., avoiding public transit) than men.

The finding that adherence to social distancing guidelines was positively associated with being less politically right-oriented fits to findings from studies recently conducted in the COVID-19 pandemic in the United States. Conway et al. (2020) argue that although much research suggests that conservatives are more sensitive to disease threats, they seem to be less concerned about the COVID-19 pandemic than liberals. However, the authors add that this ideological effect diminishes as experiences with, and the impact of the COVID-19 pandemic grows. Furthermore, our findings are supported by another recently study conducted during the COVID-19 pandemic. In this study, liberals and politically moderates show more guideline adherence than conservatives (van Holm et al., 2020). It is intuitively plausible that guideline adherence increases with acceptance of the measures. However, the moderation analysis revealed that political orientation influences the relationship between acceptance of measures and guideline adherence. This interaction effect showed that for less right-wing-orientated participants adherence to social distancing guidelines was not linked to acceptance of the measures. This link was only found in people with moderate political orientation (average values) and in people with more right-wing orientation. These findings are in line with findings from other studies in the COVID-19 context. For instance, also Capraro and Barcelo (2020) report in a recent preprint that demographic variables and political orientation are relevant characteristics in the context of protective behavior. According to their findings, being female, being older, and being left-leaning are correlated with greater intentions to wearing a face covering. Also, studies from Gollwitzer et al. (2020) and Van Bavel et al. (2020) show that supporters of right-wing political parties were less likely to adhere to protective behavior compared to liberal or left-leaning individuals.

One year after Study 1 and Study 2, the COVID-19 pandemic was still having major impact on our daily lives and causing restrictions on social contact in Germany. However, since many may also have become accustomed to these circumstances, we aimed at reinvestigating our research questions.

## Study 3 Method

As the COVID-19 pandemic progressed, restrictive measures in Germany also continued. Therefore, another goal of this research project was to investigate the research questions of the two preceding studies 1year later. For this purpose, we conducted Study 3, a replication of Study 2. Since we had no assumptions regarding changes in perception and behavioral responses to the consequences of the COVID-19 pandemic, we did not formulate explicit hypotheses and instead reexamined our research questions.

### Participants and Procedure

Our third online survey was conducted between March 15 and April 28, 2021. The recruitment of participants was as in Study 2. A total of 555 people clicked on the questionnaire. As in the previous two studies, data were excluded from participants who did not complete the questionnaire or who failed the attention check. As in the previous studies, no information about the individual characteristics of the excluded persons is available, but all participants originated from the same pool of people. The remaining sample consisted of 530 (69.4% female) employed part-time students from the same university with locations in 32 cities across Germany as in Study 1 and Study 2 (*M_age_*=26.26, *SD_age_*=4.63, *Range_age_*=19–50). As in Study 2, participants also entered the first digit of their postal code. Again, data showed that the participants came from all nine postal code areas in Germany. However again, the distribution was not equally distributed with most participants living in postal code area four where many of the locations of the university are (number of participants from area 0=8; 1=61; 2=54; 3=18; 4=165; 5=73; 6=41; 7=70; 8=77; 9=8). Fifteen of the participants reported being infected with COVID-19. Ten participants reported that they were sick, and one of those assumed that she/he was infected. All other participants reported that they were healthy. As in Study 1 and Study 2, all participants remained in the data set independent of their COVID-19 status. Participants were rewarded with course credits for their participation.

### Measures

We applied the same measures for *trust in official statistics* (Cronbach’s *α*=0.85), *political orientation*, *health anxiety* (Cronbach’s *α*=0.92), and *uncertainty tolerance* (Cronbach’s *α*=0.66) as in Study 2. This also applies to the indices self-centered prepping behavior (Cronbach’s *α*=0.76), *behavior change to avoid infection* (Cronbach’s *α*=0.78), *behavior change to not infect others* (Cronbach’s *α*=0.80), *trust in the government* (Cronbach’s *α*=0.91), *acceptance of the measures* (Cronbach’s *α*=0.89), and *adherence to the social distancing guidelines* (Cronbach’s *α*=0.64).

## Study 3 Results

As in Study 1 and Study 2, to answer RQ 1, people’s level of trust in statistics on COVID-19 from official authorities was compared and displayed in Table 1. The results show, as in the two previous studies, that trust in statistics from China was by far lowest, whereas trust in statistics from the RKI was highest.

To reinvestigate RQ 2, correlation analyses from Study 1 and Study 2 were replicated and shown in Table 8. Correlations between gender, trust in official statistics, and health anxiety with behavior variables were stronger than in the studies from 2020, whereas the link between age and political orientation with behavior change was weaker.

**Table 8:** Means, SDs, and correlations for gender, age, trust in official information, political orientation, and health anxiety with self-centered prepping behavior, behavior change to avoid infection and behavior change in order to not infect others in Study 3.

	Prepp. beh.	BC for self	BC for others
1. Gender	**0.169** ^***^	**0.110** ^*^	**0.164** ^***^
2. Age	0.072	0.043	0.018
3. Trust Info.	0.017	**0.355** ^***^	**0.278** ^***^
4. Left–Right	0.006	0.016	−0.03
5. MK-HAI	**0.261** ^***^	**0.173** ^***^	**0.103** ^*^
6. UT	**−0.115** ^**^	−0.059	−0.008
Total: *M* (*SD*)	3.20 (1.26)	3.62 (0.97)	3.62 (0.98)
Men: *M* (*SD*)	2.87 (1.28)	3.46 (0.98)	3.38 (1.01)
Women: *M* (*SD*)	3.34 (1.22)	3.69 (0.97)	3.73 (0.95)
*t*-Test	**3.92** ^***^	**2.54** ^*^	**3.79** ^***^
Cohen’s *d*	0.38	0.23	0.36

Gender: 1=male, 2=female; Trust Info.=trust in official information (Range=1–7), higher values indicate more trust; Left–Right=political orientation (Range=1–10), higher values indicate more right orientation; MK-HAI=health anxiety (Range=1–5), higher values indicate more anxiety; UT=uncertainty tolerance (Range=1–6), higher values indicate more uncertainty tolerance; Prepp. beh.= self-centered prepping behavior (Study 1: Range=0–3; Study 2: Range=1–7), higher values indicate more self-centered prepping behavior; BC for self=behavior change for self-protection/to avoid infection (Range=1–5), higher values indicate more behavior change; and BC for others=behavior change in order to not infect others (Range=1–5), higher values indicate more behavior change.

* indicates *p*<0.05;

** indicates *p*<0.01;

*** indicates *p*<0.001. Bold highlighted values are at least *p*<0.10.

For comparison reasons, the same variables as in Study 1 and Study 2 were included in multiple regressions on the dependent variables of self-centered prepping behavior, behavior change to avoid infection, and behavior change in order not to infect others (see Table 9). As in the studies from 2020, results showed that health anxiety was positively associated with self-centered prepping behavior and behavior change to avoid infection of oneself. Furthermore, in 2021, health anxiety was positively associated with behavior change in order not to infect others. Further in line with the previous studies, the results showed that trust in official statistics was positively associated with behavior change to avoid infections of oneself and others. However, in 2021, these associations were much stronger. Additionally, being female was positively associated with all behavior variables, which mirrors the pattern of Study 1 and Study 2. However, political orientation was not associated with any behavior variable in Study 3.

**Table 9:** Multiple linear regressions on self-centered prepping behavior, behavior change to avoid infection, and behavior change in order to not infect others in Study 3.

	Study 3				
	Unstandardized coefficients		Standardized coefficients		
	*B*	*SE*(*B*)	*β*	*t*	*p*
**Prepp. beh.**
(Constant)	0.38	0.47		0.80	0.421
Gender	0.48	0.11	**0.17** ^***^	4.19	0.000
Age	0.03	0.01	**0.11** ^**^	2.76	0.007
Trust Info.	0.01	0.03	0.02	0.51	0.609
Left–Right	0.02	0.03	0.02	0.67	0.499
MK-HAI	0.43	0.06	**0.26** ^***^	6.44	0.000
*adj. R^2^*	0.101				
*F*	**12.81** ^***^				0.000
**BC for self**					
(Constant)	1.06	0.35		3.00	0.003
Gender	0.27	0.08	**0.12** ^**^	3.15	0.002
Age	0.02	0.00	**0.09** ^*^	2.36	0.018
Trust Info.	0.24	0.02	**0.36** ^***^	9.12	0.000
Left–Right	0.03	0.02	0.05	1.27	0.202
MK-HAI	0.20	0.05	**0.16** ^***^	4.05	0.000
*adj. R^2^*	0.171				
*F*	**22.68** ^***^				0.014
**BC for others**					
(Constant)	1.54	0.36		4.18	0.000
Gender	0.37	0.09	**0.13** ^***^	4.12	0.000
Age	0.14	0.00	0.06	1.59	0.111
Trust Info.	0.19	0.02	**0.28** ^***^	6.97	0.000
Left-Right	0.00	0.02	0.01	0.32	0.743
MK-HAI	0.11	0.05	**0.09** ^*^	2.23	0.026
*adj. R^2^*	0.113				
*F*	**14.34** ^***^				0.000

Gender: 1=male, 2=female; Trust Info.=trust in official information (Range=1–7), higher values indicate more trust; Left-Right=political orientation (Range=1–10), higher values indicate more right orientation; MK-HAI=health anxiety (Range=1–5), higher values indicate more anxiety; Prepp. Beh.=self-centered prepping behavior (Study 1: Range=0–3; Study 2: Range=1–7), higher values indicate more self-centered prepping behavior; BC for self=behavior change for self-protection/to avoid infection (Range=1–5), higher values indicate more behavior change; and BC for others=behavior change in order to not infect others (Range=1–5), higher values indicate more behavior change.

* indicates *p*<0.05;

** indicates *p*<0.01;

*** indicates *p*<0.001. Bold highlighted values are at least *p*<0.10.

To investigate RQ 3, asking who shows more adherences to social distancing guidelines, correlations of guideline adherence with variables used in Study 2 (gender, age, trust in official statistics, political orientation, health anxiety, acceptance of the measures, and trust in government) were analyzed in a first step. The correlation matrix can be found in Table 10.

**Table 10:** Means, SDs, and correlations for gender, age, trust in official information, political orientation, health anxiety, acceptance of the measures, and trust in the government with adherence to the social distancing guidelines in Study 3.

	Guideline adherence
1. Gender	0.056
2. Age	**0.169** ^***^
3. Trust Info.	**0.179** ^***^
4. Left-Right	−0.049
5. MK-HAI	−0.002
6. Acceptance	**0.178** ^***^
7. Trust Gov.	**0.136** ^**^
Total: *M* (*SD*)	3.51 (0.82)
Men: *M* (*SD*)	3.45 (0.77)
Women: *M* (*SD*)	3.55 (0.84)
*t*-Test	1.29
Cohen’s *d*	0.12

Gender: 1=male, 2=female; Trust Info.=trust in official information (Range=1–7), higher values indicate more trust; Left-Right=political orientation (Range=1–10), higher values indicate more right orientation; MK-HAI=health anxiety (Range=1–5), higher values indicate more anxiety; Acceptance (Range=1–7)=higher values indicate more acceptance of the measures; Trust Gov.=trust in government (Range=1–7), higher values indicate more trust in the government; and Guideline Adherence=adherence to the social distancing guidelines (Range=0–5), higher values indicate more adherence.

** indicates *p*<0.01;

*** indicates *p*<0.001. Bold highlighted values are at least *p*<0.10.

As in Study 2 age, trust in official statistics and acceptance of the measures showed significant correlations with guideline adherence (the variable guidelines adherence was only collected from Study 2 onwards). For comparison reasons, the same variables as in Study 2 were included in a multiple regression on the dependent variable guideline adherence. Findings are presented in Table 11. As the correlational findings already indicated, results showed that adherence to the social distancing guidelines was positively associated with higher levels of acceptance of the measures, being of older age, and having more trust in official statistics.

**Table 11:** Multiple linear regressions on adherence to the social distancing guidelines Study 3.

	Study 3				
	Unstandardized coefficients		Standardized coefficients		
	*B*	*SE*(*B*)	*β*	*t*	*p*
**Guid. Adh.**
(Constant)	2.01	0.29		6.80	0.000
Gender	0.10	0.07	0.06	1.38	0.167
Age	0.03	0.00	**0.19** ^***^	4.51	0.000
Trust Info.	0.08	0.02	**0.15** ^**^	3.11	0.002
Left–Right	−0.02	0.02	−0.04	1.03	0.303
Acceptance	0.06	0.02	**0.10** ^*^	2.07	0.038
*adj. R^2^*	0.075				
*F*	**9.49** ^***^				0.000

Guid. Adh.=adherence to the social distancing guidelines (Range=0–5), higher values indicate more adherence, Gender: 1=male, 2=female; Trust Info.=trust in official information (Range=1–7), higher values indicate more trust; Left-Right=political orientation (Range=1–10), higher values indicate more right orientation; Acceptance=acceptance of the measures (Range=1–7), higher values indicate more acceptance.

* indicates *p*<0.05;

** indicates *p*<0.01;

*** indicates *p*<0.001. Bold highlighted values are at least *p*<0.10.

As in Study 2, we conducted moderation analyses to test whether political orientation, gender and age are moderators of the relationship between acceptance of the measures and guideline adherence. We used Hayes’ PROCESS tool (model 1). The distribution of political orientation in Study 3 was like that in Study 2. Also, here political orientation was non-normally distributed, with a skewness of 0.06 (*SE*=0.11) and kurtosis of −0.11 (*SE*=0.21), indicating a right-skewed left-leaning distribution. The average value was *M*=4.40 (*SD*=0.07) slightly below the mean of the scale, the median=5. However, results showed no moderation effect for political orientation (*p*=0.954). Moreover, neither gender (*p*=0.988), nor age (*p*=0.837) moderated the effect.

## Study 3 Discussion

Study 3 successfully replicated findings from Study 1 and Study 2: (a) Also 1year after the surveys in March and April 2020, there were still differences in trust in official statistics from different authorities: Again, data from China were believed much less than data from Europe or Germany, whereas data from the RKI were most trusted. (b) Results from all three studies showed that health anxiety increases self-centered prepping behavior and behavior change to avoid infections. Also, trust in official statistics increased behavior change to avoid infections. Regarding behavior change in order not to infect others, results in Study 3 slightly differ compared to studies 1 and 2. Whereas in the first two studies, being female, being less politically right-oriented and having trust in official statistics were positively associated with behavior change to protect others, Study 3 indicates that political orientation is no longer a relevant predictor for behavior change in order not to infect others. Moreover, neither political orientation, gender nor age showed up as moderators in Study 3. Instead, health anxiety turned out to predict behavior change in order not to infect others. This leads to the assumption that the Corona pandemic has become less an issue of political orientation than of individual characteristics related to health-related behaviors.

As Study 2, Study 3 aimed at investigating influences on adherence to social distancing guidelines. Again, results show that guideline adherence was positively associated with older age and higher acceptance of the measures. In addition, and contrary to Study 2, higher levels of trust in official information turned out to be a relevant predictor for guideline adherence, too. However, no associations were found with gender and political orientation. These findings indicate that the importance of the various predictors for guideline adherence changed as the global pandemic progressed. A relevant factor in this context may be that the levels of general acceptance with preventive measures declined substantially between the time points of studies 2 and 3. Thus, the importance of political orientation might have decreased because support for social distancing guidelines has declined in all population groups. This trend has already been suggested by Conway et al. (2020) who argue that ideological effects diminish as experiences with, and the impact of the COVID-19 pandemic grows. In contrast, trust in official information has become more relevant. This is consistent with recent findings from other studies. Bargain and Aminjonov (2020) found that higher trust was associated with decreased mobility related to non-necessary activities. Fridman et al. (2020) report that higher levels of trust in government information sources are positively related to adherence to social distancing.

## General Discussion

Today, there are numerous psychological studies on the COVID-19 pandemic context. However, many of these studies focus on screening for negative (mental health) effects of the COVID-19 pandemic. The aim of our studies was to capture early and later perceptions and behavioral reactions to the COVID-19 pandemic. Our three studies give insights into three important dimensions in the context of the COVID-19 pandemic: (a) trust in official statistics, (b) behavior change, and (c) adherence to social distancing guidelines. There are striking differences in participants’ trust in official statistics depending on whether the source is China or the RKI. The high level of trust in statistics from the RKI in our three samples corresponds with results from COSMO - Covid-19 Snapshot Monitoring a German Corona Monitor (COSMO COVID-19 Snapshot Monitoring, 2020). The weekly monitoring survey results from March 3, 2020, to April 21, 2020, show that trust in the RKI was consistently very high, even higher than trust in the German Federal Ministry of Health, the Federal Government and the WHO. However for 2021, results from Betsch (2021) show that trust in general (in government and in authorities) has declined somewhat. Furthermore, the present findings show that trust in the official statistics is a predictor of behavior change and guideline adherence. Therefore, effort should be made to ensure that trust in the data is maintained, especially in contexts where long-term measures are required, like the COVID-19 pandemic.

Health anxiety was linked to self-centered prepping behavior and behavior change to reduce personal risk in all three studies. These findings are not only intuitively plausible but also supported by other studies showing that anxiety is linked to safety behavior (e.g., Erceg et al., 2020). Our analyses also revealed bidirectional effects regarding health anxiety and prepping behavior (in Study 1–3) and between health anxiety and behavior change to avoid own infection (Study 1–3). Behavior change in order not to infect others was only associated with health anxiety in study 3. This is in line with research from Olatunji et al. (2011) and emphasizes the importance of further research in the context health anxiety. Age was not or only negligibly associated with self-centered prepping behavior. This is in line with findings from the German Corona Monitor regarding panic buying (waves 1, 2, and 3: Betsch et al., 2020a,b,c). However, gender seems to be relevant when it comes to behavior change to avoid personal and other person’s risks. In all three studies, women reported higher values on the behavior change variables (both to avoid own infection and to protect others) then men. Previous research has shown that women are more safety-oriented (Lermer et al., 2016b), especially in the health domain (Thom, 2003; Lermer et al., 2016a). Women also tend to behave generally more pro-socially (Archer, 1996) than men. Our findings imply that these observations also apply during the COVID-19 pandemic.

Results from the present study (samples 2 and 3) indicate a positive effect of acceptance of measures and trust in the government, a moderate positive effect of trust in official statistics and a small negative effect of being more politically right-wing oriented (Study 2) on adherence to social distancing guidelines. Betsch et al. (2020d) report in their Corona Monitor that German acceptance of the measures had risen sharply since mid-March 2020 and then decreased somewhat, with some fluctuations, until April 2021 (Betsch, 2021). However, overall acceptance of most of the measures was still at a high level. Our study is line with these findings. Our results reveal that approximately 1year after the outbreak of the Coronavirus pandemic, the adherence to official guidelines regarding social distancing declined somewhat. Research has shown that trust in authorities is an important factor for the acceptance of environmental measures (Zannakis et al., 2015) and adherence to health guidelines (Gilles et al., 2011; Prati et al., 2011; Quinn et al., 2013; Sibley, 2020). Political decision-makers and officials should therefore use approaches that underpin trust to promote the acceptance of measures, especially in view of long-term challenges like the COVID-19 pandemic. In particular, the pandemic should be politized at any point of time. Rather authorities as well as media and press should focus on a communication that promotes trust. Betsch et al. (2020d) recommend transparent communication and the emphasis on jointly achieved successes.

Adherence to social distancing guidelines was higher among people who were older, female, less right-wing orientation, and more accepting of the measures (Study 2). Betsch et al. (2020d) also reported small positive effects of age and (marginally significant effect) of being female on safety behavior (i.e., using face covering) in the context of the COVID-19 pandemic. Further analyses showed that the association between acceptance of the measures and guideline adherence was moderated by political orientation (Study 2). It should be noted that the variable political orientation was not normally distributed but slightly right-skewed left-leaning distributed. However, low values (1 *SD* below mean) can be interpreted as more left-wing oriented, average values (mean) as neutral and high values (1 *SD* above mean) as more right-wing oriented. Thus, the results can be interpreted as follows: for politically left-wing-oriented participants acceptance of the measures had no effect on their guideline adherence, whereas data from politically neutral and right-wing-oriented participants showed a positive link between acceptance of the measures and guideline adherence. Interestingly, the antecedents of social distancing changed over the course of a year. Gender and political orientation no longer predicted adherence to guidelines in Study 3, while trust in government became more relevant. These findings are particularly important for the current COVID-19 pandemic and for future considerations in dealing with pandemics. Obviously, the importance of political orientation decreased as the Coronavirus pandemic progressed. From a practical perspective, policymakers should periodically review and challenge their assumptions about the public’s perception of the pandemic situation. In this way, communication of the necessary measures can be adjusted in the best possible way. Here, it is of particular importance to maintain the trust of the public, especially when support for anti-Coronavirus measures decline. In addition to general trust in the government, however, trust in the government’s competencies is especially relevant. Fancourt et al. (2020, p. 464) summarize: “Public trust in the government’s ability to manage the pandemic is crucial as this trust underpins public attitudes and behaviors at a precarious time for public health.” We see further practical implications of these study findings primarily in that the results presented here may be helpful in developing and communicating interventions. The results confirm that perceptions and behavioral responses differ in Germany, both at the onset of the COVID-19 pandemic and 1year later. As other studies (e.g., Warren et al., 2020) suggest, the government should not only ensure that trust in the government is and remains high but also consider how different groups of people are addressed in campaigns.

Today, more than ever, researchers are called upon to replicate research (Bonett, 2012; Wingen et al., 2020). This can be done by conceptual or exact replications (e.g., Stroebe and Strack, 2014). We assume that especially conceptual ones are important. That means that not that exactly same thing was done, but from the basic idea the same results are found. At the time of our data collection, it was not yet possible to foresee what the research on the COVID-19 context would be like. We very much welcome the fact that so many scientists are taking up this relevant topic. This will increase the likelihood of reducing the negative consequences of future challenges such as this pandemic. Some limitations of the study must be mentioned. All three studies were correlative cross-sectional studies. Therefore, no cause–effect relationships can be proven, and future studies should consider longitudinal studies. As in many psychological studies, our samples were convenience samples and consisted of students. However, since this institution where participants were recruited is a part-time university, the students are all employed and on average older than full-time students. Furthermore, in all studies, most of the participants were female. Women tend to perceive higher risks, show more risk-averse behavior than men (Byrnes et al., 1999; Harris and Jenkins, 2006) and are more anxious than men (Maaravi and Heller, 2020) which may have influenced the study’s results. In general, there is a high consistency between our results and those of similar studies. For example, other studies have shown that women report higher levels of social distancing than men (Pedersen and Favero, 2020; Guo et al., 2021). This is in line with our findings regarding the fact that being female is a predictor for greater adherence to social distancing guidelines and behavior change in order not to infect others. Therefore, the unequal gender distribution in our sample does not seem to have distorted the results. Nevertheless, more emphasis should be put on a balanced gender distribution in future studies. Since we asked relatively personal questions (e.g., prosocial behavior), it cannot be guaranteed that there is no social desirability bias in the data. Socially desirable responding to questionnaire items is a general problem in studies relying on self-report. Consequently, future studies should aim to replicate our research findings with more indirect measures. However, the consistency of our results with the current state of research suggests that findings can be successfully replicated. Another important limitation concerns the fact that we only measured behavioral intentions but not actual behavior. Thus, future research should focus on identifying variables that can be used to observe actual behavior. Another interesting approach for future research is to consider individualism and collectivism. The results of a recently published study analyzing data from 69 countries show that the more individualistic (vs. collectivistic) a country is, the higher the COVID-19 infection rates were (Maaravi et al., 2021). Furthermore, future studies in the COVID-19 context should investigate the influence of information sources such as social network platforms in the context of trust (Bunker, 2020; Limaye et al., 2020).

Overall, the present findings are helpful to target specific groups for preventive campaigns in the context of a pandemic. The fact that differentiated communication can be relevant is also described by Warren et al. (2020) in the COVID-19 vaccine context. A review paper by Bish and Michie (2010) conducted to identify key determinants of safety behavior in the context of the 2009 H1N1 influenza pandemic reports that being female and of older age is linked to adopting safety behaviors. This is also confirmed by the results of present studies for the COVID-19 context. In addition, trust, less right-wing political orientation, and acceptance of measures were shown to be relevant variables for safety behavior. These findings show how important it is to consider individual differences when it comes to prevention measures implemented on a large scale for the sake of a greater good.

## Data Availability Statement

The original contributions presented in the study are publicly available. This data can be found at: https://osf.io/y7hxe/.

## Ethics Statement

Ethical review and approval was not required for the study on human participants in accordance with the local legislation and institutional requirements. The patients/participants provided their written informed consent to participate in this study.

## Author Contributions

All authors developed the study concept, contributed to the study design, and interpreted the results. Material testing and data collection were performed by EL and MH. The data were analyzed by EL and MH. EL drafted the manuscript, and MH, MR, SG, and FB provided critical revisions. All authors contributed to the article and approved the submitted version.

## Conflict of Interest

The authors declare that the research was conducted in the absence of any commercial or financial relationships that could be construed as a potential conflict of interest.

## Publisher’s Note

All claims expressed in this article are solely those of the authors and do not necessarily represent those of their affiliated organizations, or those of the publisher, the editors and the reviewers. Any product that may be evaluated in this article, or claim that may be made by its manufacturer, is not guaranteed or endorsed by the publisher.
